# Supplementary material for: Resolving the full spectrum of human genome variation using Linked-Reads
Source: Genome Res. 2019 Apr;29(4):635–45. doi: 10.1101/gr.234443.118 (PMC6442396; doi:10.1101/gr.234443.118)
Supplement: Supplemental Material [file supp_gr.234443.118_Supplemental_longranger-2.2.zip › longranger-2.2.tar.bz2!/lr2.2-public/lib/stan/cmdstan/stan/doc/api/html/agrad_2rev_2matrix_2sd_8hpp_source.html]

Stan: src/stan/agrad/rev/matrix/sd.hpp Source File


|  |  |
| --- | --- |
|  | Stan  2.5.0  probability, sampling & optimization |


- Main Page
- Related Pages
- Namespaces
- Classes
- Files

- File List
- File Members

All Classes Namespaces Files Functions Variables Typedefs Enumerations Enumerator Friends Macros Pages

- src
- stan
- agrad
- rev
- matrix

sd.hpp

Go to the documentation of this file.

1 #ifndef STAN\_\_AGRAD\_\_REV\_\_MATRIX\_\_VARIANCE\_HPP

2 #define STAN\_\_AGRAD\_\_REV\_\_MATRIX\_\_VARIANCE\_HPP

3

4 #include <cmath>

5 #include <vector>

6 #include <boost/math/tools/promotion.hpp>

7 #include <stan/math/matrix/Eigen.hpp>

8 #include <stan/math/matrix/mean.hpp>

9 #include <stan/agrad/rev/var.hpp>

10 #include <stan/agrad/rev/vari.hpp>

11 #include <stan/agrad/rev/matrix/stored\_gradient\_vari.hpp>

12 #include <stan/math/error\_handling/matrix/check\_nonzero\_size.hpp>

13

14 namespace stan {

15

16  namespace agrad {

17

18  namespace { // anonymous

19

20  // if x.size() = N, and x[i] = x[j] =

21  // then lim sd(x) -> 0 [ d/dx[n] sd(x) ] = sqrt(N) / N

22

23  var calc\_sd(size\_t size,

24  const var\* dtrs) {

25  using std::sqrt;

26  vari\*\* varis = (vari\*\*) memalloc\_.alloc(size \* sizeof(vari\*));

27  for (size\_t i = 0; i < size; ++i)

28  varis[i] = dtrs[i].vi\_;

29  double sum = 0.0;

30  for (size\_t i = 0; i < size; ++i)

31  sum += dtrs[i].vi\_->val\_;

32  double mean = sum / size;

33  double sum\_of\_squares = 0;

34  for (size\_t i = 0; i < size; ++i) {

35  double diff = dtrs[i].vi\_->val\_ - mean;

36  sum\_of\_squares += diff \* diff;

37  }

38  double variance = sum\_of\_squares / (size - 1);

39  double sd = sqrt(variance);

40  double\* partials = (double\*) memalloc\_.alloc(size \* sizeof(double));

41  if (sum\_of\_squares < 1e-20) {

42  double grad\_limit = 1 / std::sqrt((double)size);

43  for (size\_t i = 0; i < size; ++i)

44  partials[i] = grad\_limit;

45  } else {

46  double multiplier = 1 / (sd \* (size - 1));

47  for (size\_t i = 0; i < size; ++i)

48  partials[i] = multiplier \* (dtrs[i].vi\_->val\_ - mean);

49  }

50  return var(new stored\_gradient\_vari(sd, size,

51  varis, partials));

52  }

53

54  }

55

63  var sd(const std::vector<var>& v) {

64  stan::math::check\_nonzero\_size("sd(%1%)", v,"v",(double\*)0);

65  if (v.size() == 1) return 0;

66  return calc\_sd(v.size(), &v[0]);

67  }

68

69  /\*

70  \* Return the sample standard deviation of the specified vector,

71  \* row vector, or matrix. Raise domain error if size is not

72  \* greater than zero.

73  \*

74  \* @tparam R number of rows

75  \* @tparam C number of columns

76  \* @param[in] m input matrix

77  \* @return sample standard deviation of specified matrix

78  \*/

79  template <int R, int C>

80  var sd(const Eigen::Matrix<var,R,C>& m) {

81  stan::math::check\_nonzero\_size("sd(%1%)", m,"m",(double\*)0);

82  if (m.size() == 1) return 0;

83  return calc\_sd(m.size(), &m(0));

84  }

85

86  }

87 }

88

89 #endif

vari.hpp

stan::agrad::memalloc\_

memory::stack\_alloc memalloc\_

**Definition:** var\_stack.cpp:16

stan::agrad::sum

fvar< T > sum(const Eigen::Matrix< fvar< T >, R, C > &m)

**Definition:** sum.hpp:14

stan::agrad::sqrt

fvar< T > sqrt(const fvar< T > &x)

**Definition:** sqrt.hpp:15

mean.hpp

stored\_gradient\_vari.hpp

var.hpp

stan::math::mean

boost::math::tools::promote\_args< T >::type mean(const std::vector< T > &v)

Returns the sample mean (i.e., average) of the coefficients in the specified standard vector...

**Definition:** mean.hpp:23

stan::agrad::sd

var sd(const std::vector< var > &v)

Return the sample standard deviation of the specified standard vector.

**Definition:** sd.hpp:63

Eigen.hpp

stan::memory::stack\_alloc::alloc

void \* alloc(size\_t len)

Return a newly allocated block of memory of the appropriate size managed by the stack allocator...

**Definition:** stack\_alloc.hpp:168

stan::agrad::var

Independent (input) and dependent (output) variables for gradients.

**Definition:** var.hpp:27

stan::math::e

double e()

Return the base of the natural logarithm.

**Definition:** constants.hpp:86

stan::math::size

int size(const std::vector< T > &x)

**Definition:** size.hpp:11

stan::agrad::variance

var variance(const std::vector< var > &v)

Return the sample variance of the specified standard vector.

**Definition:** variance.hpp:51

check\_nonzero\_size.hpp

stan::math::check\_nonzero\_size

bool check\_nonzero\_size(const char \*function, const T\_y &y, const char \*name, T\_result \*result)

Return true if the specified matrix/vector is of non-zero size.

**Definition:** check\_nonzero\_size.hpp:33


---

[ Stan Home Page ]

*© 2011–2014,
Stan Development Team.*
